# A Case Report of Acute Severe Necrotizing Pancreatitis following the Johnson & Johnson Vaccine against the Novel SARS-CoV-2

**DOI:** 10.1155/2023/9965435

**Published:** 2023-03-22

**Authors:** Ayrton I. Bangolo, Mahabuba Akhter, Auda Auda, Rahina Akram, Vignesh K. Nagesh, Donnee Athem, Reenu Thomas, Ligaya Tibalan, Mansi Trivedi, Saima Mushtaq, Neha Singh, Pracheta Bagale, Georgemar V. Arana, Tayyaba Khan, Shelja Sharma, Swetha Mynedi, Dhara D. Patel, Mandeep Saini, Madhurya R. Chinthakuntla, Kareem Ahmed, Mary Gad, Srikara Dheer D. R. Gondhi, Georgemar Arana, Rohini B. Gurumoorthy, Simcha Weissman

**Affiliations:** ^1^Department of Internal Medicine, Palisades Medical Center, North Bergen, NJ, USA; ^2^Department of Internal Medicine, University of Washington, Seattle, Washington, USA

## Abstract

Acute pancreatitis is an inflammatory condition, which is a leading gastrointestinal cause of hospitalization in the United States. Several conditions are associated with acute pancreatitis. More recently, there have been a few cases reported of acute pancreatitis following the Pfizer-BioNTech COVID-19 mRNA vaccine. To our knowledge, no cases of acute pancreatitis have been yet reported following the Johnson & Johnson's Janssen COVID-19 vaccine (J& J vaccine). Herein we report a 34-year-old male with no significant past medical history admitted with acute necrotizing pancreatitis, the day following the receipt of the J&J vaccine. Based on the Naranjo and the modified Naranjo scale, the patient met the requirements for probable drug induced pancreatitis. This case report has the objective to raise awareness of a potentially severe side effect of the J&J vaccine. We hope to use this case to support screening all patients for previous history of acute pancreatitis before administration of the J& J vaccine.

## 1. Introduction

Acute pancreatitis is an acute inflammatory process of the pancreas with a mortality rate of 17 percent in patients who develop pancreatic necrosis [1]. About two-thirds of acute pancreatitis cases are associated with gallstones and chronic alcohol use disorders [2]. Acute pancreatitis following vaccinations, such as the Human Papillomavirus vaccine has been previously reported in the literature [3]. More recently, a few cases of acute necrotizing pancreatitis have been linked to the Pfizer-BioNTech COVID-19 mRNA vaccine [4, 5]. No cases of acute necrotizing pancreatitis associated with the Johnson& Johnson's Janssen COVID-19 vaccine (J&J vaccine) have yet been reported in the literature. Herein we report the case of a young, nonalcoholic male who presented with severe abdominal pain, the day following receipt of the J&J vaccine. He was admitted with acute necrotizing pancreatitis and an extensive work up did not yield an alternative etiology. This case report highlights a potentially severe side effect of the J&J vaccine. We propose that administration of the J&J vaccine is a risk factor for acute necrotizing pancreatitis.

## 2. Case Report

This is a 34-year-old male with no significant past medical history and not taking any medications who presented for evaluation of a 1-day history of severe constant epigastric pain, radiating to the back. He reported associated nausea with 2 episodes of nonbloody, nonbilious vomiting, subjective fever, and chills. Of note, the patient received the J&J vaccine the day prior to presentation. He denies any tobacco use, and reports drinking on social occasions. His last drink was 2 weeks prior to the presentation. He denies any previous similar episodes or any association with food. Of note, prior to vaccination administration, the patient tested negative for COVID-19 by reverse transcription-polymerase chain reaction.

**Figure 1:**
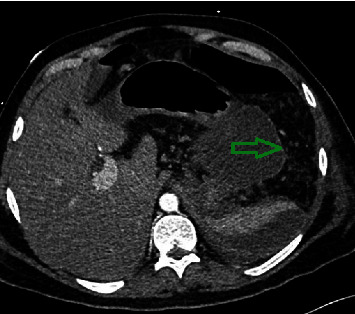
Computed tomography of the abdomen and pelvis showing necrotizing pancreatitis (green arrow).

On physical exam, he was found to be tachycardic, diaphoretic, and febrile with a temperature of 100.9 Fahrenheit. The abdomen was tender in all 4 quadrants, more so in the epigastric area. His laboratory results revealed leukocytosis, elevated lipase, and elevated liver enzymes as seen in Table 1. The computed tomography (CT) of the abdomen and pelvis was consistent with acute necrotizing pancreatitis (Figure 1). The right upper quadrant ultrasound showed a patent portal vein and no signs of acute cholecystitis. Additional laboratory studies to further investigate the etiology of acute pancreatitis were within normal limits (Table 1).

The patient was admitted to the Intensive Care Unit, started on Lactated Ringer and pain medications. Infectious disease and gastroenterology were consulted, and the patient was started on empiric meropenem. The patient's hospital course was complicated with ileus and duodenal perforation for which he underwent an emergent exploratory laparotomy. He was discharged after 30 days of hospitalization.

## 3. Discussion

Acute pancreatitis is an inflammatory condition of the pancreas characterized by abdominal pain and elevated levels of pancreatic enzymes. Several conditions are associated with acute pancreatitis. Gallstones and chronic alcohol use disorders are the etiology for most cases [6]. Several vaccines have been reported in the literature as potential etiology of acute pancreatitis [3, 7, 8]. More recently, a few cases of vaccine induced necrotizing pancreatitis have been reported with the Pfizer-BioNTech COVID-19 mRNA vaccine [4, 5]. The J&J vaccine has been associated with a few adverse events, among which deep-vein thrombosis [9]^*∗*^. The Naranjo scale was developed to help standardize assessment of causality for all adverse drug reactions including vaccines [10]. Our patient is a healthy young male, with no other risk factors for acute pancreatitis, and with symptoms onset the day following the receipt of the vaccination. He scored 5 on the Naranjo scale, consistent with probable association of necrotizing pancreatitis with the vaccine.

Increased alkaline phosphatase and total bilirubin levels have been historically used in prediction of biliary pancreatitis [11]. However, a study by Bradley and Salam found hyperbilirubinemia in up to 47 percent of patients with acute pancreatitis without biliary obstruction [12]. Paralytic ileus has been reported in the literature as a complication of acute pancreatitis [13]. Spontaneous bowel perforation can be a rare and life-threatening complication of acute necrotizing pancreatitis [14]. Our patient had significant hyperbilirubinemia, although the liver and biliary imaging did not reveal any stones or signs of obstruction. His pancreatitis was complicated with ileus and duodenal perforation which are rare complications. We encourage clinicians to include these complications in the differential of any worsening clinical course of acute pancreatitis.

The mainstay of management of a patient with acute pancreatitis consists of supportive care with fluid resuscitation, pain control, and nutritional support [15]. Clinical signs of infection and abdominal imaging demonstrating the presence of gas within the necrosis should prompt the use of antibiotics [16]. Our patient had a fever documented on admission, leukocytosis, and pancreatic necrosis on abdominal imaging. It was appropriate to initiate antibiotics.

The main limitation of our report is that no other cases have reported this association. Although this is the first of its kind report, widely validated scores such as the Naranjo and the modified Naranjo scores were used to establish the association. Thus, we are confident that our report is accurate.

## 4. Conclusion

This case report serves to contribute to the growing body of the literature regarding the potential adverse effects of the novel J&J vaccine against SARS-CoV2. By encouraging more clinicians to report similar cases of necrotizing pancreatitis, we can help identify a patient population in which this vaccine should be avoided. In addition to current guidelines, we suggest a questionnaire be developed to further screen patients at risk for acute pancreatitis.

## Figures and Tables

**Table 1:** Laboratory values on admission.

	Laboratory values (normal values)
White blood cells	**18.9** × **10** *∗* **3** (4.8–10.8 × 10 *∗* 3) microliter (*μ*L)
Neutrophils	**86%** (45–70)
Blood urea nitrogen	**45** (7–25) milligram per deciliter (mg/dL)
Creatinine	**2.19** (0.7–1.30) mg/dL
Calcium	**7.3** (8.6–10.3) mg/dL
Albumin	3.5 (3.5–5.7) gram per deciliter (g/dL)
Aspartate aminotransferase	91 (13–39) unit per liter (U/L)
Alanine aminotransferase	85 (7–52) U/L
Alkaline phosphatase	48 (34–104) U/L
Triglycerides	**260** (<150) mg/dL
Total bilirubin	**9.9** (0.3–1) mg/dL
Ceruloplasmin	35 (18–36) mg/dL
Lipase	**1,026** (11–82) U/L
Antiliver/kidney microsomal antibodies	<=20 (<=20)
Immunoglobulin G	846 (600–1640) mg/dL
Antinuclear antibody	Negative
Actin antibodies	<20 (≤20) units (U)
Antimitochondrial antibody	Negative
C-reactive protein	**20 millimeter per hour (0**–**15)**

Abnormal lab values are in bold.

## Data Availability

All data generated or analyzed during this study are included within the article.
